# Supplementary material for: Evaluation of Acute and Subacute Toxicity and LC-MS/MS Compositional Alkaloid Determination of the Hydroethanolic Extract of Dysphania ambrosioides (L.) Mosyakin and Clemants Flowers
Source: Toxins (Basel). 2022 Jul 12;14(7):475. doi: 10.3390/toxins14070475 (PMC9316831; doi:10.3390/toxins14070475)

toxins

Supplementary file

Evaluation of Acute and Subacute Toxicity and LC-MS/MS Compositional Alkaloids Determination of the Hydroethanolic Extract of *Dysphania ambrosioides* (L.) Mosyakin and Clemants Flowers

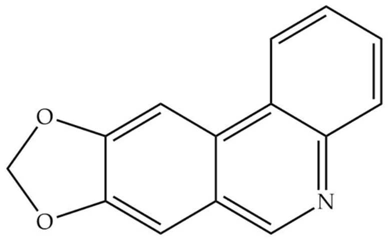

Compound CID : 443684
Compound number : 1
Trisphaeridine

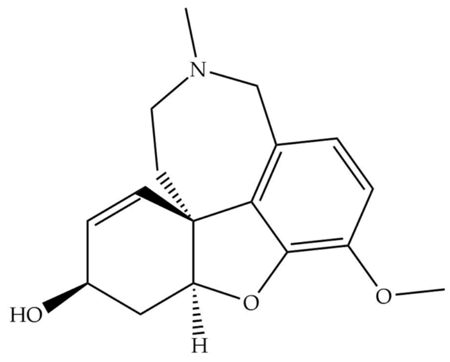

Compound CID : 9651
Compound number : 2
Galanthamine

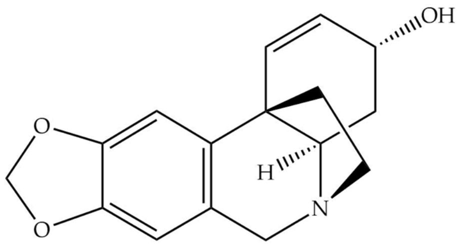

Compound CID : 398937
Compound number : 3
Crinine

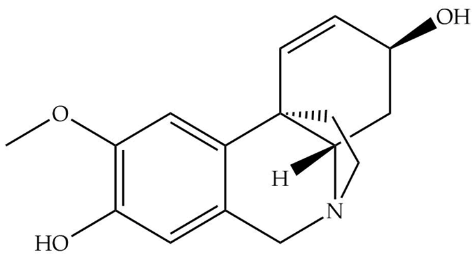

Compound CID : 443683
Compound number : 4
Demethylmaritidine

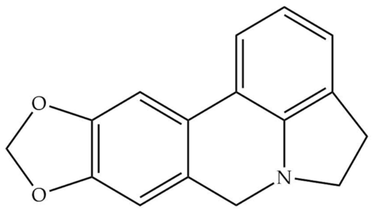

Compound CID : 619567
Compound number : 5
Anhydrolycorine

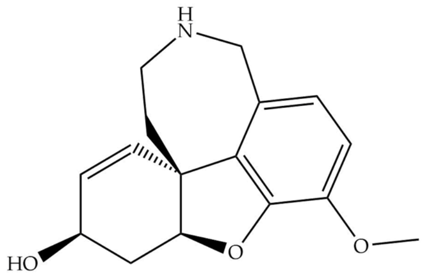

Compound CID: 9838394
Compound number : 6
Norgalanthamine

Figure S1. Identified alkaloids found in *D. ambrosioides*' flowers hydroethanolic extract, along with their compound CID (retrieved from PubChem on May 10, 2022).

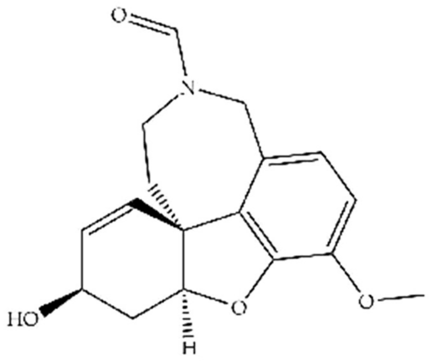

Compound CID : 22899516
Compound number : 7
N-formylgalanthamine

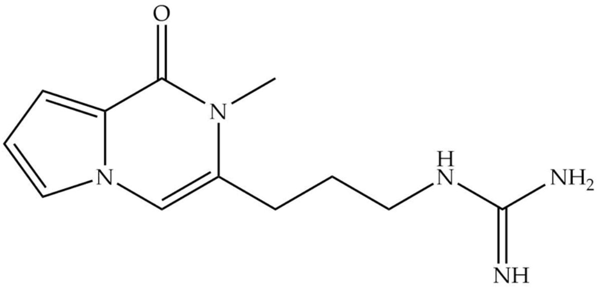

Compound CID : 114748
Compound number : 8
Peramine

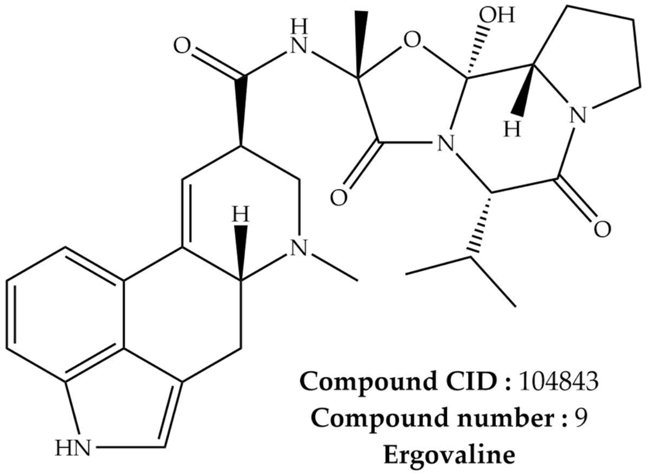

Compound CID : 104843
Compound number : 9
Ergovaline

Figure S1. *Cont.*

Table S1. Screening of alkaloids from *D. ambrosioides* hydroethanolic extract, and standards molecules using LC-MS/MS.

<table>
<tr><th>Compound</th><th>[M-H]+ literature</th><th>Selected m/z</th></tr>
<tr><td>Ergovaline</td><td>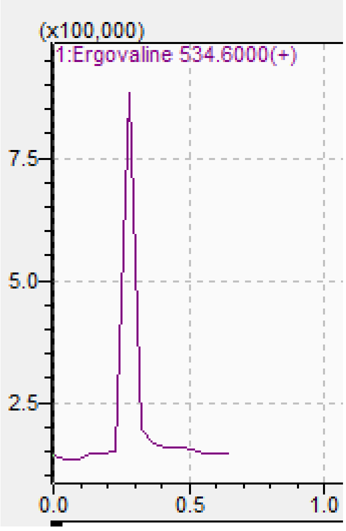
</td><td>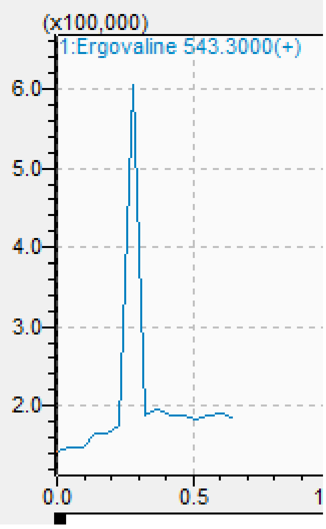
</td></tr>
<tr><td>Galanthamine</td><td>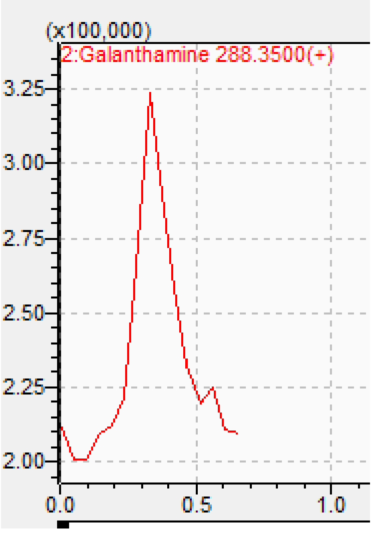
</td><td>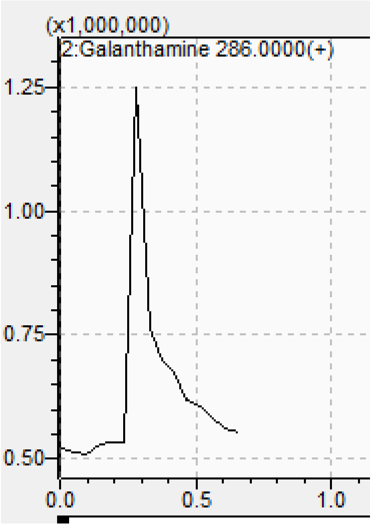
</td></tr>
<tr><td>Norgalanthamine</td><td>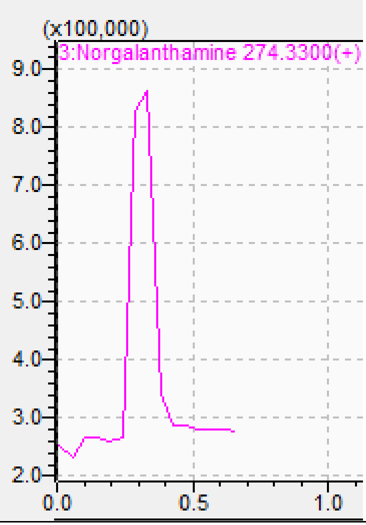
</td><td>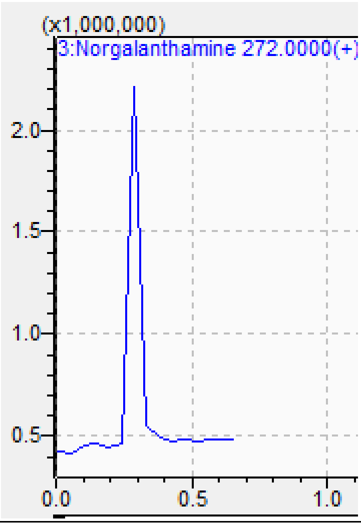
</td></tr>
</table>

<table>
<tr><td>Anhydrolycorine</td><td>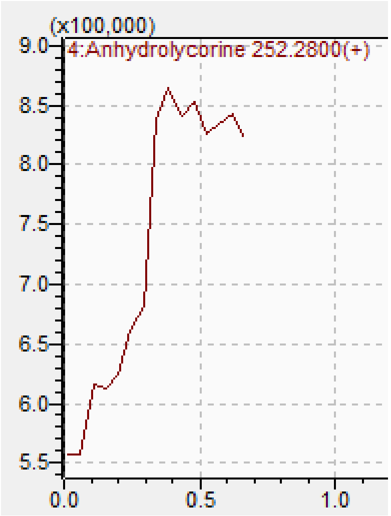
</td><td>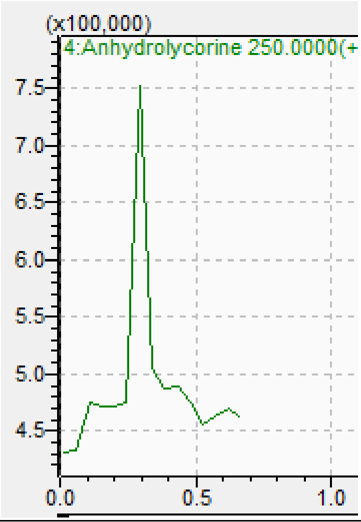
</td></tr>
<tr><td>Trisphaeridine</td><td>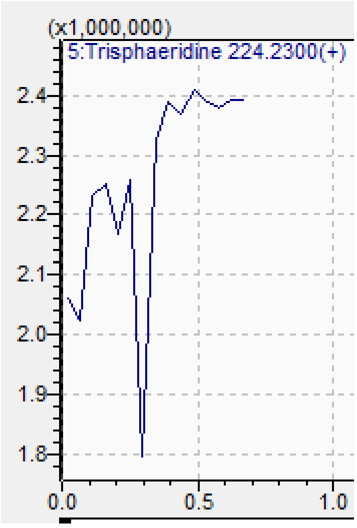
</td><td>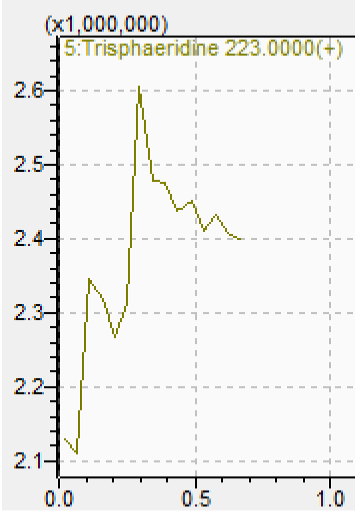
</td></tr>
<tr><td>Crinine</td><td>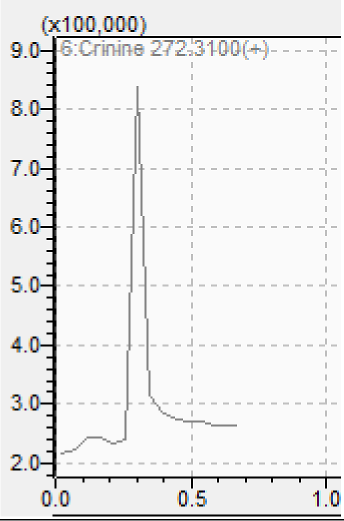
</td><td>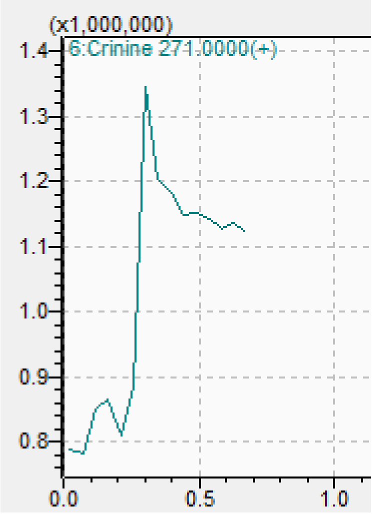
</td></tr>
</table>

Demethylmaritidine

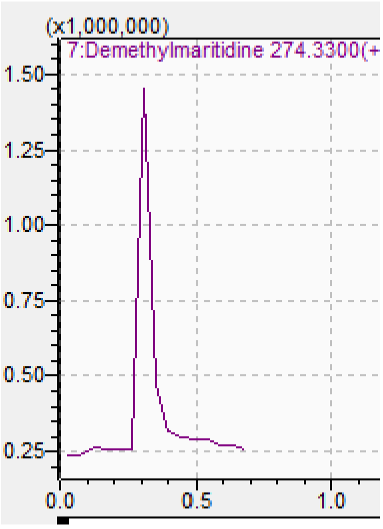

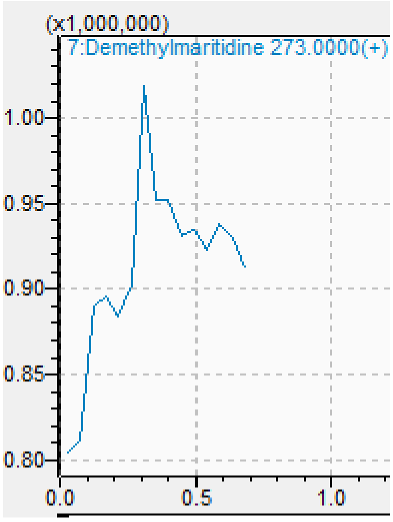

N-formylnorgalanthamine

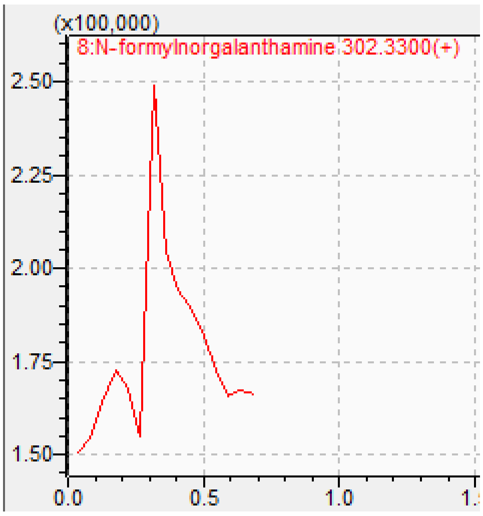

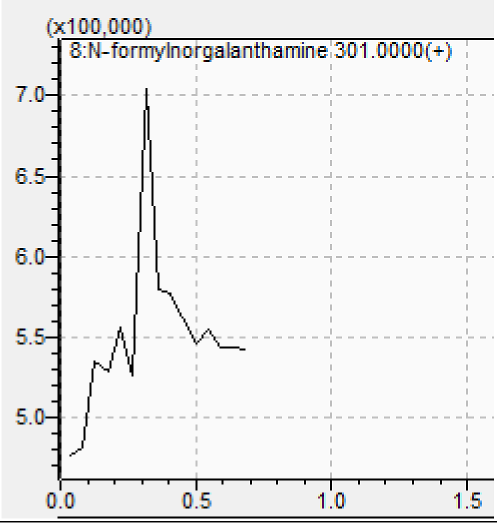

Peramine

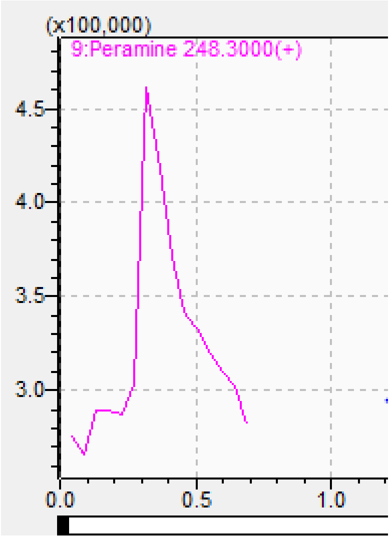

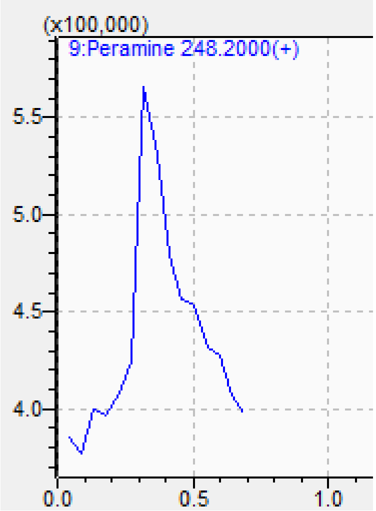

Supplement: Supplementary file 1 [file toxins-14-00475-s001.zip › toxins-1746401-supplementary.pdf]
